# Cerebral Microbleed Automatic Detection System Based on the “Deep Learning”

**DOI:** 10.3389/fmed.2022.807443

**Published:** 2022-03-24

**Authors:** Pingping Fan, Wei Shan, Huajun Yang, Yu Zheng, Zhenzhou Wu, Shang Wei Chan, Qun Wang, Peiyi Gao, Yaou Liu, Kunlun He, Binbin Sui

**Affiliations:** ^1^Department of Radiology, Beijing Tiantan Hospital, Capital Medical University, Beijing, China; ^2^National Clinical Research Center for Neurological Diseases, Beijing, China; ^3^Tiantan Neuroimaging Center of Excellence, Beijing, China; ^4^Department of Neurology, Beijing Tiantan Hospital, Capital Medical University, Beijing, China; ^5^Beijing Institute for Brain Disorders, Beijing, China; ^6^Laboratory of Translational Medicine, Chinese PLA General Hospital, Beijing, China; ^7^Key Laboratory of Ministry of Biomedical Engineering and Translational Medicine, People’s Liberation Army General Hospital, Beijing, China

**Keywords:** cerebral microbleed, deep learning, neural network, segmentation, clinical evaluation

## Abstract

**Objective:**

To validate the reliability and efficiency of clinical diagnosis in practice based on a well-established system for the automatic segmentation of cerebral microbleeds (CMBs).

**Method:**

This is a retrospective study based on Magnetic Resonance Imaging-Susceptibility Weighted Imaging (MRI-SWI) datasets from 1,615 patients (median age, 56 years; 1,115 males, 500 females) obtained between September 2018 and September 2019. All patients had been diagnosed with cerebral small vessel disease (CSVD) with clear cerebral microbleeds (CMBs) on MRI-SWI. The patients were divided into training and validation cohorts of 1,285 and 330 patients, respectively, and another 30 patients were used for internal testing. The model training and validation data were labeled layer by layer and rechecked by two neuroradiologists with 15 years of work experience. Afterward, a three-dimensional convolutional neural network (CNN) was applied to the MRI data from the training and validation cohorts to construct a deep learning system (DLS) that was tested with the 72 patients, independent of the aforementioned MRI cohort. The DLS tool was used as a segmentation program for these 72 patients. These results were evaluated and revised by five neuroradiologists and subjected to an output analysis divided into the missed label, incorrect label, and correct label. The interneuroradiologists DLS agreement rate, which was assessed using the interrater agreement kappas test, was used for the quality analysis.

**Results:**

In the detection and segmentation of the CMBs, the DLS achieved a Dice coefficient of 0.72. In the evaluation of the independent clinical data, the neuroradiologists reported that more than 90% of the lesions were directly detected and less than 10% of lesions were incorrectly labeled or the label was missed by our DLS. The kappa value for interneuroradiologist DLS agreement reached 0.79 on average.

**Conclusion:**

Based on the results, the automatic detection and segmentation of CMBs are feasible. The proposed well-trained DLS system might represent a trusted tool for the segmentation and detection of CMB lesions.

## Introduction

Cerebral microbleeds (CMBs) are radiological constructs that were first observed and defined on MRI (1). T2*-weighted gradient-recalled echo (GRE) and susceptibility-weighted imaging (SWI) are commonly used to detect CMB in clinical practice (2). On GRE images or SWI, a CMB is a small elliptical or circular lesion of 2–5 mm but sometimes up to 10 mm (3). According to previous studies, SWI is usually the main modality recommended for quantifying numbers of CMBs, as it shows higher sensitivity and reliability for CMB detection than GRE imaging. The pathophysiology of CMB has not yet been fully elucidated. Histopathologically, microbleeds represent the perivascular focal collection of hemosiderin deposits (1–5). Vitreous degeneration of small vessels and vascular amyloidosis are considered to be the two main pathological mechanisms. They might damage the small vascular wall and cause the destruction of the blood-brain barrier. The focal remnant deposits of hemosiderin are most likely secondary to such arteriolar and capillary damage caused by multiple mechanisms, which result in blood product leakage in the perivascular space (6). A group of risk factors for CMB has been reported, including age, hypertension, cholesterol, diabetes mellitus, and smoking (7–9). CMB is associated with an increased risk of several diseases and conditions. CMBs increase the risk of subsequent ischemic stroke and intracranial hemorrhage (ICH) (2, 3, 10, 11). CMBs are associated with small vascular disease (SVD) and are thus more likely to accompany strokes with lacunar infarction than infarction caused by cardioembolism or atherosclerosis (12). CMB is also expected to cause ICH (8, 13). Therefore, CMB was considered a predictor of future stroke and hemorrhage in patients receiving thrombolytic therapy or long-term antithrombotic treatment for ischemic stroke (3).

Cerebral microbleeds (CMBs) are also associated with an increased risk of cognitive impairment and dementia in patients with normal cognitive function, mild cognitive impairment, and dementias such as Alzheimer’s disease (4, 14–16). In addition, CMBs may be present in individuals with some genetic diseases, such as cerebral autosomal dominant arteriopathy with subcortical infarcts and leukoencephalopathy (CADASIL) or Moyamoya disease (17, 18). The risk and extent of CMB, which is considered a biomarker of SVD, has been used as an index for evaluating the status of underlying diseases and might influence the management of these diseases (12). Thus, a systemic and quantitative evaluation of CMB with high accuracy and efficiency is essential in assessing disease prognosis. At present, visual scoring systems are used in CMB evaluations, including the Microbleed Anatomical Rating Scale (MARS) (19) and the Brain Observer MicroBleed Scale (BOMBS) (20). However, the reliability of these methods in assessing the number and location of CMBs is relatively low without the use of evaluation tools. In recent years, automated or semiautomated brain imaging analysis methods have been applied to evaluate CMBs (21–23). A deep learning system (DLS) for automatic CMB detection was developed and analyzed in terms of reliability to support clinical work with consistent and efficient CMB identification and simplify the clinical workflow of CMB marking. We invited five clinical neuroradiologists to assess the performance of the proposed DLS, especially the number and location of CMBs based on SWI sequences. This study aimed to validate an appropriately trained DLS that could be trusted by a neuroradiologist with sufficient experience.

## Materials and Methods

### Standard Protocol Approvals, and Patient Consent

This study was approved by the ethics committee of Beijing Tiantan Hospital and fulfilled the Declaration of Helsinki.

### Image Dataset

We retrospectively obtained MRI-SWI data with good SWI image quality from 1,615 patients, and all the data were obtained from Beijing Tiantan Hospital. According to the SWI acquisition protocol used clinically, scans were obtained using multiple different scanners with a field strength of 1.5T or 3T. In this dataset, we labeled 10,525 lesions, including 9,387 small size lesions ranging in size from 2 to 5 mm and 1,138 large lesions ranging in size from 5 to 10 mm. The basic information of the patients and manufacturers is provided in Table 1, and more detailed information about lesion sizes is presented in Table 2. The clinical evaluation dataset (test data) included MRI-SWI images from 72 patients with CMBs in the Third China National Stroke Registry (CNSR-III), a nationwide registry of ischemic stroke or transient ischemic attack (TIA) in China based on etiology, imaging, and biological markers that recruit consecutive patients with ischemic stroke or TIA from 201 hospitals that cover 22 provinces and four municipalities in China. This dataset is independent of the previous 1,615 patients.

**TABLE 1:** Basic information of the patients, manufacturers, and parameters of scanners.

Patients characteristics (training/validation dataset)	Patient (images) metric
Number of patients	1285/330
Female to male ratio	405:880/95:235
**Different macufacturers (numbers in the training/validation datasets)**	
GE	287/72
Siemens	356/88
Philips	642/170
**Field strength (numbers in the training/validation datasets)**	
1.5T	174/34
3T	1111/296
**Scanner model (numbers in the training/validation datasets)**	
Verio	89/25
Ingenia	429/116
Achieva	77/13
Trio Tim	110/33
Signa HDxt	71/10
DiSCOVERY MR750	205/70
Ingenia CX	133/33
Skyra	16/2
Avanto	100/18
Aera	43/6
SIGNA Explorer	12/2
Prisma	0/2
**Resolution (numbers in the training/validation datasets)**	
512 × 384	96/15
432 × 432	459/157
512 × 512	326/60
256 × 192	183/47
256 × 232	62/15
480 × 480	21/7
768 × 624	9/3
256 × 224	42/9
224 × 256	10/2
320 × 320	9/1
384 × 264	3/0
320 × 260	18/3
640 × 520	13/2
310 × 320	1/0
352 × 352	9/1
256 × 256	21/8
260 × 320	1/0
560 × 560	2/0

**TABLE 2:** Data distribution.

	patients	Small lesions	Large lesions
Training dataset	1,285 (79.6%)	7,461 (79.5%)	927 (81.5%)
Validation dataset	330 (20.4%)	1,926 (20.5%)	211 (18.5%)
Summary	1,615	9,387	1,138

### Data Quality Control

Minor artifacts or mildly reduced signal-noise ratios with no effects on diagnosis or no artifacts and optimal artifacts were selected for the evaluation of image quality in this retrospective study. Diagnostic Screening: All patient electric health records (EHRs) were reviewed and reanalyzed by medical doctors before preprocessing the images, labeling, and generating ensemble models. The segmentation labels with CMBs were based on a manual slice by slice analysis of the MRI-SWI data. After labeling all images with CMBs, all data were rechecked and endorsed by two radiologists with 15 years of clinical experience, which were used for DLS training and validation.

### Network Architecture

The SWI image is a three-dimensional (3D) axial slice, and the image size is Z*X*Y. Z represents the number of slices and X*Y represents the length and width of each slice. Microbleeding is a disease with contextual information. The normal network used two-dimensional (2D) U-net and 3D U-net for prediction, but the number of slices of the SWI was quite different. If 3D U-net and resampling are used, some slice information will be missing, and a simple 2D U-net will lack the upper and lower slice information. Therefore, in this article, we adopted a new approach. In the training process, three consecutive layers of slices were used as input. The size was X*Y*3, and the output was X*Y. The middle slice of the positive sample had microbleeds, and the middle slice of the negative sample had no microbleeds. Bleeding was not set for the analysis of whether microbleeds were present in the upper and lower layers. Here, the ratio of positive to negative was 1:1. During the test, the entire image was input into the network in sequence according to the scanning order.

**FIGURE 1:**
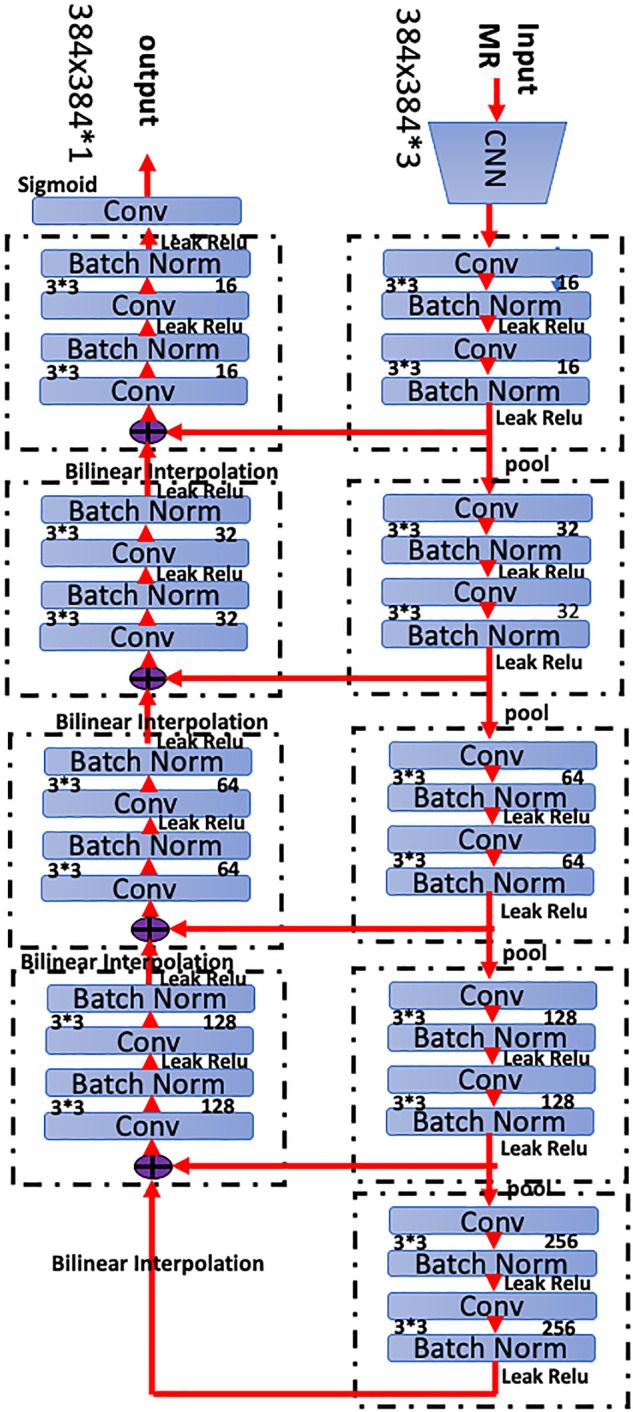
Network architecture of the proposed three-dimensional (3D) convolutional neural network (CNN). The network has 28 layers integrating six residual blocks. Bilinear interpolating arrows indicate upsampling operations to provide dense predictions for the segmentation task. Skip connections are used to fuse low- and high-level features in the network. Batch normalization is a linear transformation of the features to reduce covariance shift and accelerate the training process. The convolution bar represents the convolution operation that computes features. The number *64* indicates the number of channels in that layer, and *3* 3 *3* 3 *3* denotes the size of the 3D CNN kernels.

We implemented a 3D CNN to extract representative features for complicated CMBs based on the MRI-SWI sequences. Specifically, we designed a full CNN architecture composed of encoder and decoder paths to conduct the segmentation task. More specifically, our network was based on the widely-used modified 3D U-Net architecture with 3 layers. The detailed network architecture is shown in Figure 1, which provides a detailed description of the segmented network used to detect CMBs.

### Establishing the Deep Learning Algorithm

Before feeding the model, all MRI-SWI data were preprocessed by scaling the global (3D) image intensities and were standardized across the acquisition parameters to increase the convergence rate of network training. We performed the normalization and alignment based on the histogram peaks to the white matter content in the MRI. All images were cropped into squares according to the shortest side, and the size was cropped or resized to 384 × 384 pixels. According to the histogram, we deleted fewer points (less than 1e-4), and the window width was determined and then max-min normalized. Lesions with sizes other than 2–10 mm were deleted.

The training set and validation set consisted of CMB data (*n* = 1,285 positive volumetric scans and 330 positive volumetric scans, respectively). The model was trained using 3D axial SWI slices. The SWI data from all patients were preprocessed, resized, and normalized to have a uniform size of 384 × 384 × 3 pixels and pixel intensities in the range of 0–1. Using these data, the network was trained using binary cross-entropy loss and the Adam optimizer with an initial learning rate of 10^–3^. During training, model training progress was monitored using a validation set Dice score. The learning rate was reduced by a factor of 0.1. An early stopping criterion was implemented if the validation Dice did not improve for 30 consecutive epochs to avoid model overfitting. Training stopped if the validation Dice score did not improve for 60 consecutive epochs. At the end of the training, the model with the highest Dice score for the validation set was retrieved. Its performance was evaluated on the test dataset. The training was stopped when the training loss was less than 10 and the validation scores reached 0.8, as we presumed that the DLS reached the optimal performance at this time. The dataset was augmented in the training process, including image vertical, rotation, translation, contrast changes and other parameters, to increase the robustness of the model. Thus, it forms a more diverse dataset with slight differences.

If the prediction mask and the reference mask intersected, a value greater than the threshold value was considered predicted correctly; otherwise, prediction error was considered. Similarly, if the reference mask and forecast masks intersect, a value greater than the threshold value was considered correctly predicted; otherwise, the prediction miss was considered. The threshold was set to 0.4 obtained from the optimized model results. Computed precision and recall were analyzed using this method. After the establishment of the model, 90 healthy patients without CMBs were used as the control to test the model, and no false-positive CMBs were detected. Then, the model entered the evaluation phase.

### Evaluation Dataset and Reference Standard

Commonly used metrics known as the Dice score, precision, recall, and accuracy were used to evaluate the performance of the proposed segmentation networks (24). Pixel level dice score was the primary model performance criteria and it was calculated as follows:


TP=pixel|Pixelscorrectlypredictedaspositive|

=|P⁢r⁢e⁢d⁢i⁢c⁢t⁢e⁢d⁢M⁢a⁢s⁢k∩G⁢r⁢o⁢u⁢n⁢d⁢t⁢r⁢u⁢t⁢h⁢m⁢a⁢s⁢k|

FP=pixel|Pixelswronglypredictedaspositive|

FN=pixel|Pixelswronglypredictedasnegative|

$$D\,i\,c\,e_{p\,i\,x\,e\,l}=\frac{2*T\,P_{p\,i\,x\,e\,l}}{2*T\,P_{p\,i\,x\,e\,l}+F\,P_{p\,i\,x\,e\,l}+F\,N_{p\,i\,x\,e\,l}}$$

Along with the dice score, the pixel level precision and recall were also calculated as follows:


$$P\,r\,e\,s\,i\,c\,i\,o\,n_{p\,i\,x\,e\,l}=\frac{T\,P_{p\,i\,x\,e\,l}}{T\,P_{p\,i\,x\,e\,l}+F\,P_{p\,i\,x\,e\,l}}$$

$$R\,e\,c\,a\,l\,l_{p\,i\,x\,e\,l}=\frac{T\,P_{p\,i\,x\,e\,l}}{T\,P_{p\,i\,x\,e\,l}+F\,N_{p\,i\,x\,e\,l}}$$

Also, along with the pixel-level computations, to understand how good the model is in identifying isolated lesions, the lesion level precision and recall were computed. For this computation, first, the individual lesions were identified as a set of continuous positive pixels from both the predicted and ground truth mask. Next, the overlap between the predicted lesions and the ground truth lesions was computed and the lesions were termed as TP lesions if this overlap was greater than 40% of the true lesion. Following this, the lesion-level precision and recall were computed as follows:


TP=lesion|Correctlypredictedaslesions|

Predicted=lesion|Predictedlesions|

True=lesion|Groundtruthlesions|

$$P\,r\,e\,s\,i\,c\,i\,o\,n_{l\,e\,s\,i\,o\,n}=\frac{T\,P_{l\,e\,s\,i\,o\,n}}{{\text{Predicted}}_{l\,e\,s\,i\,o\,n}}$$

$$R\,e\,c\,a\,l\,l_{l\,e\,s\,i\,o\,n}=\frac{T\,P_{l\,e\,s\,i\,o\,n}}{T\,r\,u\,e_{l\,e\,s\,i\,o\,n}}$$

We count the data level and patient level at the same time, and the data level is calculated on the entire data set. The patient level is calculated for each patient first and then averaged.

Furthermore, based on the condition of whether the model was able to identify at least one correct lesion, the patients were also classified into the TP, TN, FP, and FN categories. Based on this data, the patient-level FP rate (FPR), FN rate (FNR), and TP rate (TPR) were calculated as follows:


$$F\,P\,R=\frac{F\,P}{T\,N+F\,P}$$

$$F\,N\,R=\frac{F\,N}{T\,P+F\,N}$$

$$T\,P\,R=\frac{T\,P}{T\,P+F\,N}$$

The FP rate indicates the model’s tendency for wrongly identifying a patient as having infarction (Type I error rate) whereas, FNR indicates the possibility of the model missing a patient with infarction (Type II error rate). Based on the FPR and TPR, the receiver operating characteristics (ROC) curve was constructed: the abscissa was FPR and the ordinate was TPR. Then the area under the ROC (AUC) was calculated. Pixel-level ROC and lesion-level ROC were defined as follows:

Pixel-level ROC: taking each pixel as a sample, the ROC curve is calculated from the pixel prediction probability and ground truth.

Lesion-level ROC: taking each lesion as a sample, the average pixel probability of each lesion is counted, and the ROC curve is calculated from the average probability and ground truth.

Compared with pixel-level ROC, lesion-level ROC is more clinically relevant. So we constructed the lesion-level ROC to evaluate the performance of the system.

We generated predictions for 72 patients randomly chosen by a doctor among patients who had SWI sequences in their records from the CNSR-III research group to evaluate the performance of the model and assess whether it would meet the clinical requirements. The clinical diagnosis must meet the inclusion criteria, and all of these patients are independent of the previous training and validation datasets.

The clinical doctors included in this study are top experts neuroradiologists with at least 15 years of clinical experience. After DLS prediction, we asked them to categorize the prediction results into three subgroups: correct label, missed label, and incorrect label. Each of these terms was defined as follows:

Correct label: the label was accurate compared with the ground truth.

Missed label: compared with the ground truth, the model did not produce the corresponding label.

Incorrect label: the model assigned additional labels that were not in the ground truth label during the test.

Ground truth: two different chief physicians double confirmed the ground truth label in the test dataset (72 patients).

Doctors were requested to perform the segmentation to the best of their abilities, without any constraint on time or duration to ensure that they evaluated the data in its best state. They revised the prediction results obtained from DLS when the prediction results were missing or incorrect.

### Statistical Analysis

The clinical evaluation was performed by five clinical medical doctors to assess the deep learning segmentation results. The interradiologist agreement test was performed for each validation case using the SPSS software (version 20.0) (IBM, Armonk, NY, United States). The statistical significance was set to *P* < 0.05, and a kappa value >0.21, based on the ground truth.

## Results

### Patient Demographic Characteristics

**FIGURE 2:**
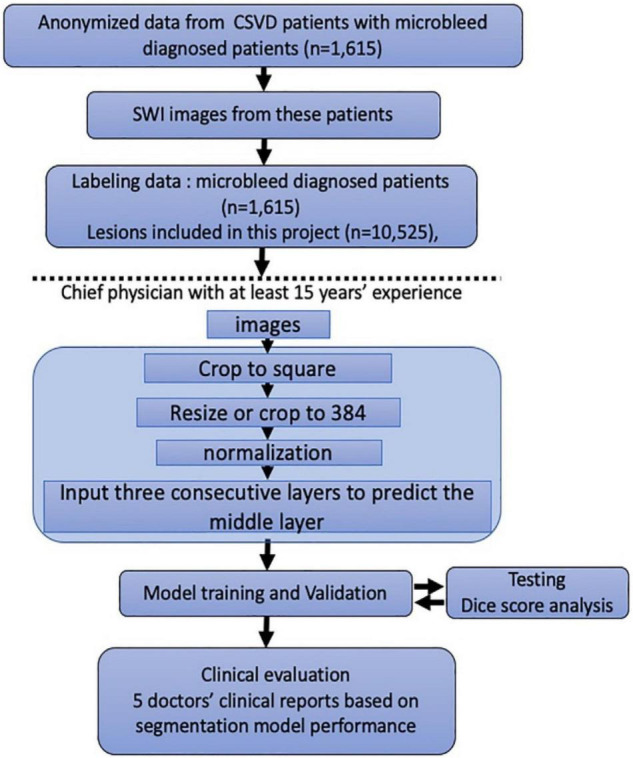
Flowchart of the patients’ distribution in training and clinical evaluation sets. Model training and clinical evaluation steps use the distribution and classification of all samples in each step.

In Figure 2, we present the entire method for the DLS setup. A total of 1,615 MRI examinations with l0,525 lesions identified in 1,615 patients were included. We randomly distributed these data into a training cohort and a validation cohort; thus, no significant differences in sex or age were observed.

Additionally, our dataset comprised an adequate number of CMBs, and the distribution of the lesion data had no bias. The parameters of these data were obtained from a similar investigator and scanner. We confirmed the scanner parameters of pixel and thinness. The brightness and contrast were normalized before being input into the DLS system.

### DLS Set-Up and Performance of the DLS Contouring Method

**FIGURE 3:**
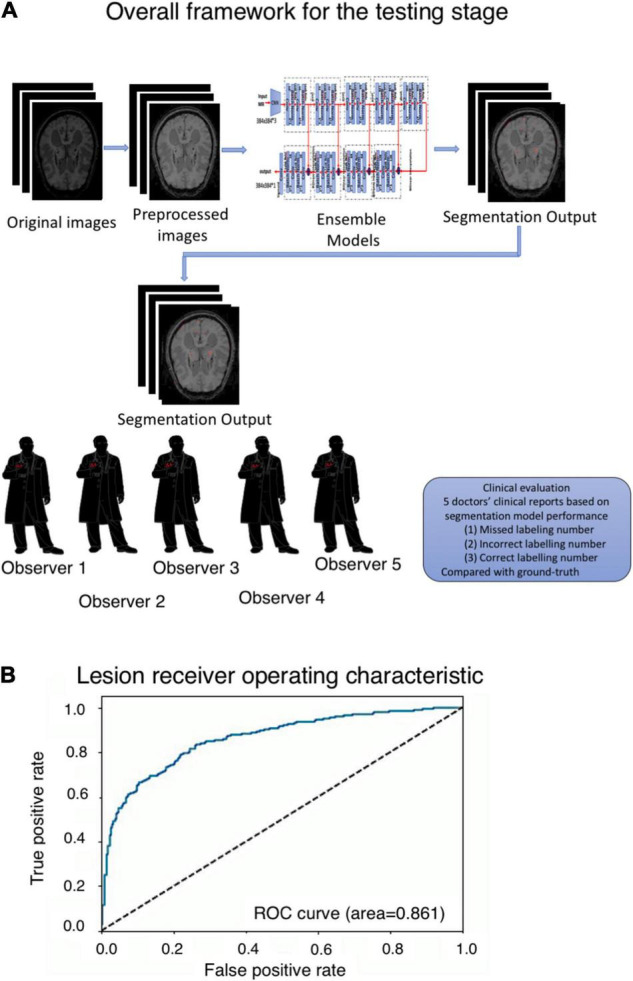
**(A)** Overall framework for the testing stage. **(B)** Segmentation receiver operating characteristic (ROC) curve and area under the curve (AUC) score in the lesion level analysis.

A total of 10,525 lesions were manually labeled to establish the DLS. Briefly, we manually labeled approximately 9,387 small size lesions (2–5 mm, 7,461 lesions for the training set and 1,926 lesions for the validation set) and 1,138 large lesions (5–10 mm, 927 lesions for the training set, and 211 lesions for the validation set) for training and validation (Table 1). The network architecture of the proposed 3-dimensional convolutional neural network is shown in Figure 1, and more detailed information about the network is presented in the methods section. After training and validation, the DLS was tested using the testing dataset. The average pixelwise DSC, precision, and recall of the proposed DLS reached 0.72, 0.718, and 0.765, respectively. The 3D lesionwise precision and recall reached 0.751 and 0.852, respectively. A lesion level analysis was performed on the independent test set, and the results showed that the sensitivities of detecting the small and large lesions were 84.4 and 93.51%, respectively. Additionally, in the lesion level analysis, the AUC score of the proposed DLS system was 0.861, and the ROC curve is shown in Figure 3B. The patient level analysis was also performed with the FP, FN, TN, and TP of 10, 2, 84, and 93, respectively. And the FP rate and FN rate were 0.106 and 0.021, respectively. The detailed results of the model are presented in Table 3.

**TABLE 3:** Model performance obtained from the testing dataset.

	DSC	Precision	Recall	Sensitivity	Specificity
Small lesions	0.71	0.707	0.762	84.4%	78.07%
Large lesions	0.73	0.729	0.768	93.51%	83.72%
In average	0.72	0.718	0.765	/	/

The data show the relabeling results after a comparison between the DLS tool and expert labeling results. From the data, we found that the labels attained from the model were accurate and perfectly matched the contour of the real signal. However, the labeling tools and pixels did not adequately control the manual labeling, and the Dice score did not adequately reflect the DLS segmentation results. These data could only support DLS training and validation. Visually, we examined all the data and found that our DLS and human experts had strong consistency in the lesion contour, but the Dice score was low, as described above.

### Assessment of DLS-Generated Contours by Human Experts

Figure 3A presents the overall framework for data prediction and the clinical evaluation process. Based on the labeling sensitivity and Dice score, the sensitivity of labeling small lesions was approximately 84.47% and that of large lesions was approximately 93.51%, with an average Dice score of approximately 0.72. The specificity of labeling was approximately 78.07% for small lesions and 83.72% for large lesions (Table 3).

**FIGURE 4:**
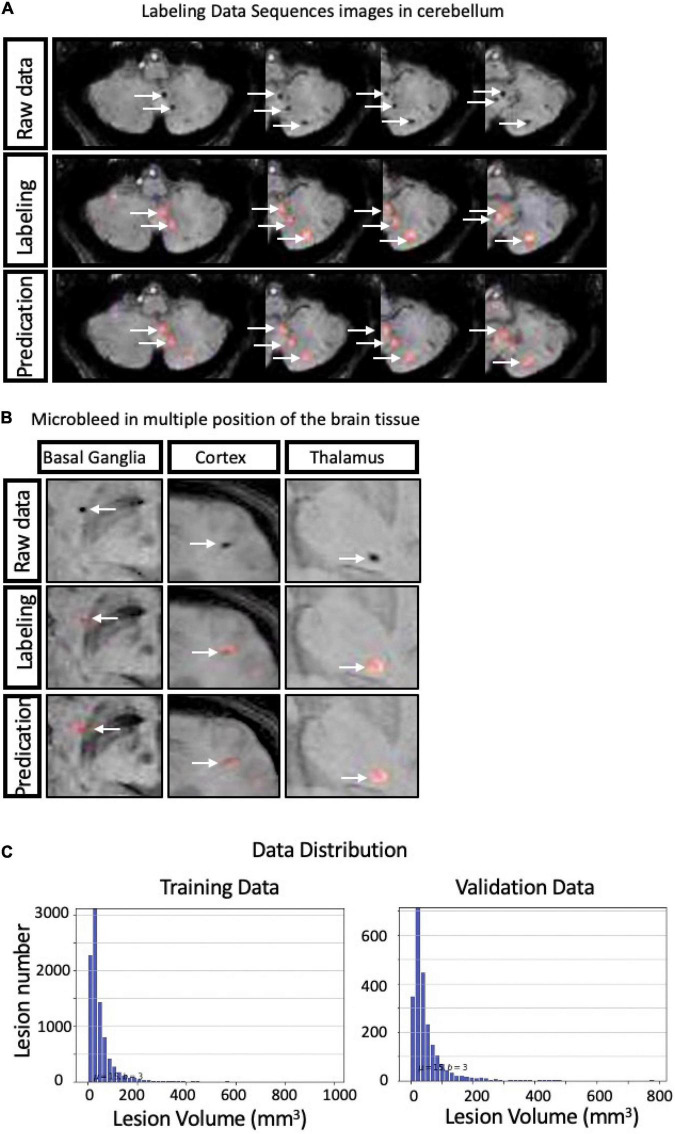
Representative cases of manual cerebral microbleeding (CMB) labeling and labeling with the deep learning system (DLS) system **(A,B)** along with the data distribution **(C)**.

However, in the clinical evaluation, the doctors evaluated the output labeling results and revised the labeling results to “missed label,” “incorrect label,” and “correct label.” The missed label group included approximately 20.6 lesions on average and 2.5% in total, and the incorrect-label group included approximately 35.6 lesions on average and 4.3% in total. We concluded that using DLS as a contouring accuracy evaluation criterion is reliable and provides accurate lesion quantification. The average kappa value for the internal agreement between observers and DLS prediction was 0.79. Detailed information in the clinical evaluation is presented in Table 4. Several examples obtained from the DLS are shown in Figure 4 and compared with those obtained manually.

**TABLE 4:** Clinical evaluation.

	Observer 1	Observer 2	Observer 3	Observer 4	Observer 5	Average
Correct label	787 (94.4%)	770 (92.8%)	790 (94.6%)	784 (93.3%)	761 (91.2%)	778.4 (93.3%)
Incorrect label	27 (3.2%)	44 (5.3%)	24 (2.9%)	30 (3.6%)	53 (6.4%)	35.6 (4.3%)
Missed label	20 (2.4%)	16 (1.9%)	21 (2.5%)	26 (3.1%)	20 (2.4%)	20.6 (2.5%)

## Discussion

Cerebral microbleed (CMB) is closely related to many diseases, including SVD, AD, and CADASIL. A previous study has shown that in addition to the location of the CMB, the number is also an independent predictor of the severity of cognitive impairment and dementia in multiple fields (25). Therefore, systematic and accurate quantification of CMB is of great clinical significance.

Based on the SWI data from 1,615 patients with a total of 10,525 lesions, we established a DLS that automatically and objectively segmented CBMs. Compared with other studies (26–28) that automatically recognized CMBs using deep convolutional networks, our DLS was trained with a larger dataset, and the sensitivity and specificity of the model were high, suggesting that it was reliable and would better serve clinicians. Previous studies usually adopted 2D CNNs to construct automatic detection systems, but they lacked upper and lower slice information. In our study, the FN rate of the DLS was low, and we used the widely-used modified 3D U-Net architecture. It made full use of the spatial information of biomarkers and accelerated the computing speed. In addition to studies that used 2D/3D CNNs to detect CMBs on MRI-SWI, Chesebro et al. (29) presented an algorithm for microbleed automated detection using geometric identification criteria (MAGIC) to detect CMBs automatically. It has reasonable precision on both T2*-weighted GRE images and SWI and had high sensitivity in longitudinal identification, with 50% of longitudinal microbleeds correctly labeled. Limited to the algorithm, this study was unable to discriminate between edge artifacts and true positives better than other studies using deep convolutional networks.

We evaluated the DLS performance based on the Dice score, which allows for minor uncertainties in the neighborhood of a few pixels, and the region-wise F1 score, which may not be a suitable indicator for success in evaluating lesions. The AUC of our DLS was 0.861, revealing the excellent performance of the system. Due to the high AUC and low FP rate and FN rate, we propose that it accurately quantified CMBs. Based on a manual data recheck and the variation in lesion marking by individual neuroradiologists, we performed a clinical evaluation based on a multicenter analysis with a score scale. Our DLS performed favorably according to the evaluation by neuroradiologists with an average accuracy of 93.3%. Our marking results were directly or clinically accepted, with most DLS-identified CMBs agreed upon by expert specialists. Moreover, these processes were performed much faster than the manual evaluation process [DLS 2.8 s/case vs. doctors 146 s/case (on average)], which is time-consuming and produces systemic and quantified results, significantly minimizing heterogeneity among neuroradiologists in the delineation of lesions. The results in this study showed that our model was used for the diagnosis and evaluation of CMBs and was more reliable than manual evaluation performed by specialists.

### Limitations

First, several different MRI devices with varying scan parameters produced all the images evaluated in this study. The use of these images might increase the data diversity in training the algorithm and testing interpretation subjectivity. However, we were unable to include all the different devices or their corresponding parameter sets for each patient. Therefore, the further clinical application of our system may be challenging due to this limitation. Second, due to the heterogeneity in different neuroradiologists’ clinical backgrounds, the accurate recognition and consistent interpretation of the number and location of CMBs by all of these clinicians was challenging. Notably, all annotations made in the dataset have been endorsed by associate chief physicians with at least 15 years of experience. As our model was trained on these data, the limitation of clinical experience in these doctors might affect their evaluation of CMBs and subsequently affect the training process. Improving data quality using more experienced doctors and rigorous training of study protocols might optimize the reliability of training our DLS model. Third, the well-trained DLS has advantages in overcoming the heterogeneity of individual human interpretations with good consistency based on the training features (30). This automated procedure is independent of clinical experience, overcoming limitations imposed by an individual physician’s visual sensitivity and clinical experience. The results from the DLS report are produced instantly by the graphical processing unit after input with the output scanning results, which is helpful for neuropathologists to perform the interpretation process faster. Prospective clinical studies are needed to determine whether this hypothesis is valid, and the interpretation should also be modified by performing a post-DLS analysis to match the equipment and the DLS. In addition, our DLS system for CMBs is based only on MRI-SWI and does not include other useful clinical diagnostic information, such as natural history and other imaging performance in the resulting output. Thus, the information is limited in producing a powerful and clinically significant prediction, and differential diagnosis, such as calcification and normal vascular fluid voids, is sometimes needed. Currently, our DLS only serves as a method for assisting neuroradiologists. Future studies involving more comprehensive clinical information are necessary. Another limitation of this study is that it is based on local and regional data. All the data were collected in China and thus do not include data from other countries and regions.

## Conclusion and Contributions

In summary, we developed a DLS tool to perform the CMB lesion segmentation. Our results show that DLS can significantly and quickly masks CMBs in less time to reduce physicians’ repetitive labor. Additionally, based on the DLS model, variation within and between neuroradiologists might be reduced. The resulting output produced by the system will be more subjective.

## Data Availability Statement

The original contributions presented in the study are included in the article/supplementary material, further inquiries can be directed to the corresponding author.

## Ethics Statement

The studies involving human participants were reviewed and approved by Beijing Tiantan Ethics Committee. According to the requirements of national legislation and institutions, written informed consent for participation was not required for this study.

## Author Contributions

PF, WS, and HY wrote the initial draft of the manuscript. WS prepared figures and made preliminary revisions. YZ and ZW contributed to DLS development and medical test organizations. QW, ZW, SWC, and KH performed the preliminary revision. YL, KH, and BS performed crucial revisions. All authors together planned the manuscript, critically revised the initial draft, and made final improvements prior to submission.

## Conflict of Interest

The authors declare that the research was conducted in the absence of any commercial or financial relationships that could be construed as a potential conflict of interest.

## Publisher’s Note

All claims expressed in this article are solely those of the authors and do not necessarily represent those of their affiliated organizations, or those of the publisher, the editors and the reviewers. Any product that may be evaluated in this article, or claim that may be made by its manufacturer, is not guaranteed or endorsed by the publisher.
